# Laparoscopic resection of a large adrenal myelolipoma: a case report

**DOI:** 10.1186/1757-1626-2-9313

**Published:** 2009-12-11

**Authors:** Supriyo Ghatak, Asit Ranjan Mridha

**Affiliations:** 1 Department of Surgical Gastroenterology, School of Digestive and Liver Diseases, Institute of Postgraduate Medical Education and Research, 244 A J C Bose Road, Kolkata- 700 020, India; 2 Department of Gastrointestinal Pathology, School of Digestive and Liver Diseases, Institute of Postgraduate Medical Education and Research, 244 A J C Bose Road, Kolkata- 700 020, India

## Abstract

A 53 year man presented with pain right upper quadrant for seven months. On evaluation he was found to have a large right adrenal myelolipoma. Laparoscopically by transperitoneal right flank approach this was removed. Postoperative recovery was uneventful. Operative specimen's histopathological examination confirmed the diagnosis of adrenal myelolipoma.

## Introduction

Adrenal myelolipoma is a rare benign tumour of the adrenal. Most of the time these tumours are asymptomatic. When they are symptomatic they usually present with pain abdomen. Surgery is indicated when the tumour is symptomatic or there is suscpicion of malignancy. Traditionally laparoscopic removal of adrenal tumour of more than 5 cm-6 cm size is contraindicated [1]. Here we removed an 11 cm myelolipoma by laparoscopy without complications.

## Case presentation

**Figure 1:**
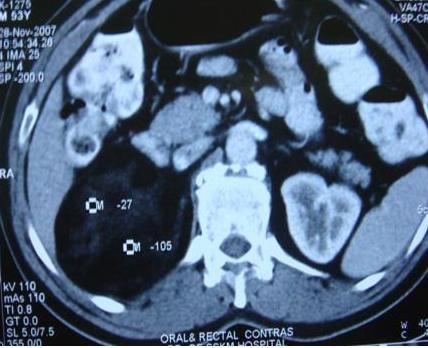
**Axial section CT scan of the right sided adrenal myelolipoma**.

**Figure 2:**
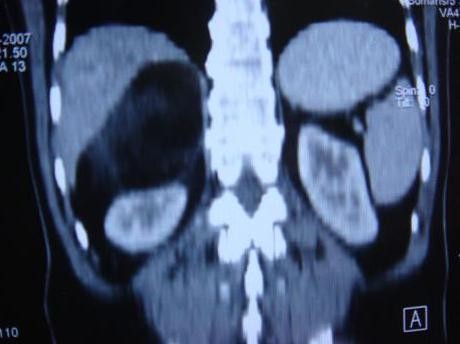
**Coronal section CT scan of right sided adrenal myelolipoma**.

**Figure 3:**
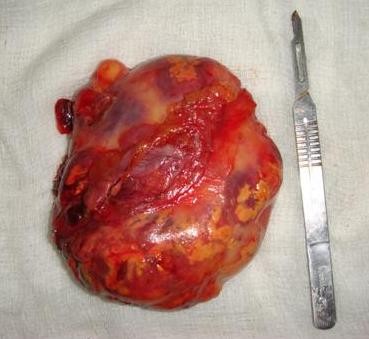
**Resected adrenal myelolipoma**.

**Figure 4:**
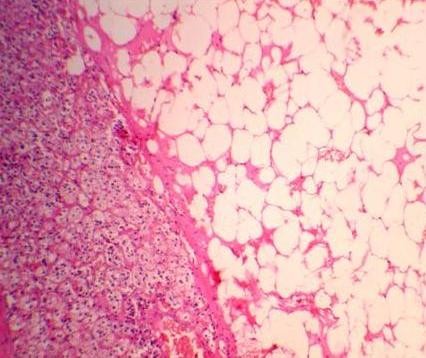
**(Hematoxylin & Eosin, × 40) Mature adipose tissue peripherally surrounded by adrenal cortical cells**.

**Figure 5:**
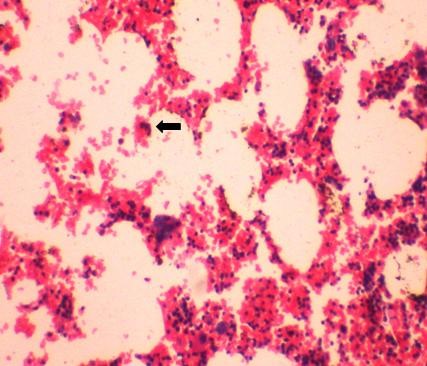
**(Hematoxylin & Eosin, × 100) Mature adipose tissue with hemopoetic cells including megakaryocytes**. Black arrow: megakaryocyte.

This 53-year-old Indo-Aryan man of Indian nationality presented to us with the symptoms of dull aching pain in the right upper quadrant of the abdomen for seven months. There is no history of fever, vomiting, gastrointestinal bleeding. There is no significant past medical or family history. He was detected to have both diabetes mellitus and hypertension during evaluation. Which were easily brought under control with oral hypoglycaemic agent (glimepiride 2 mg once daily) and anthypertensive (ramipril 5 mg once daily). Physical examination was unremarkable. Ultrasonography revealed a hyperechoic SOL (9.9 × 7.7 cm) in the right supra-renal region. CT scan showed a well defined rounded patchily hypodense lesion (8.43 cm × 7.69 cm) with discrete capsule containing fat density areas seen at right adrenal area, adjacent fat plane was maintained. The Hounsfield unit of the fat containing areas was -105 (Figure 1 and Figure 2). Right kidney has been pushed downwards by the tumour. His complete blood counts, liver function tests and chest X-ray were normal. Urinary metanephrine and serum potassium levels were normal. He was taken up for laparoscopic adrenalectomy as the tumour was symptomatic as well as large in size. Surgery was done in left lateral position. First port (10 mm) was made by open technique in right anterior axillary line just below the costal margin. After creating the pneumoperitoneum three more ports were made, one 5 mm, 5 cm behind the first port, one 10 mm, 5 cm in front of the first port and one 5 mm close to the midline below the costal margin for retracting the liver. After exploration subhepatic peritoneum is divided and right lobe of liver retracted upwards with a liver retractor introduced through the extreme left port. There were few adhesions between the left surface of the tumour and the inferior vena cava (IVC), these were divided with hook diathermy. Left border of IVC was cleared caudally upto right renal vein. Right adrenal vein was dissected out, clipped and cut. Inferior adrenal artery was found to be arising from the right renal artery, which was also clipped and cut. But no significant sized superior and middle adrenal artery was found. After the whole gland was mobilized it was taken out through a right flank incision of 6 cm. The specimen size was 11 cm × 10.5 cm and weighed 405 grams (Figure 3). One 24 Fr tube drain was placed in the right subhepatic space. Postoperative recovery was uneventful. Drain was removed after 24 hours. Patient was discharged from the hospital on day 6. Microscopic examination showed predominantly mature adipose tissue interspersed with extramedullary hematopoietic components of myeloid, erythroid and megakaryocyte series suggestive of adrenal myelolipoma (Figure 4 and Figure 5). Post surgery the patient is doing well after 12 months.

## Discussion

Myelolipomas are well-circumscribed lesions that contain mature adipose tissue intermixed with mature myeloid elements. The adrenal gland is the most common site, but myelolipomas also occur rarely in extra-adrenal locations [2]. The etiology still remains unknown. Some reports suggested endocrine dysfunction can be the etiology of adrenal and extra-adrenal myelolipoma. Cushing's syndrome, Addison's disease, adrenal hyperplasia, and chronic use of exogenous steroids have been reported in association with myelolipoma [2-4]. Injecting pituitary extract subcutaneously into experimental rats can induce myelopoiesis [2,5]. Metaplasia of the reticulo-endothelial cells of blood capillaries in the adrenal gland in response to stimuli such as infection, stress or necrosis has been proposed in pathogenesis of myelolipomas [6].

Myelolipomas of the adrenal and extra-adrenal gland are usually asymptomatic but larger lesions can cause symptoms from mass effect or haemorrhage [6-9]. Symptomatic tumours need surgical resection. They are often found incidentally on radiographic studies [10]. Autopsy studies have reported the incidence of myelolipomas as 0.2% in the general population [11]. Characteristic appearances on CT scan and MRI scan of adrenal myelolipoma exclude extensive metabolic workup or surgical exploration [12]. The Hounsfield Unit (HU) is valuable in this regards as it quantify the pixel value of a CT scan area so that the radiologist can compare the composition of one tissue with that of another [13]. The lesion is typically seen as a well-encapsulated, heterogeneous suprarenal mass of low density (less than -30 HU equivalent to mature fat) interspersed by denser myeloid tissue on the CT scan [14]. Large myelolipomas may be confused with necrotic adrenal carcinoma [12]. Other differential diagnoses are retroperitoneal liposarcoma and leiomyosarcoma [12]. It has been found that adrenal malignant neoplasms are usually larger than 6 cm [12]. So the recommendation for incidentally detected asymptomatic adrenal tumour of more than 6 cm is surgical removal [12]. In this case the Hounsfield Unit of the lesion on CT scan was suggestive of myelolipoma, but his tumour was symptomatic as well as larger than 6 cm.

If laparoscopic expertise is available then the excision is done laparoscopically. Previously it was thought to be a contraindication for laparoscopic approach when the tumour size exceeds 5 cm - 6 cm. But several recent studies have shown that laparoscopic approach is technically feasible, safe and comparable with open approach in these patients [15,16].

## Consent

Written informed consent was obtained from the patient for publication of this case report and accompanying images. A copy of the written consent is available for review by the Editor-in-Chief of this journal.

## Competing interests

The authors declare that they have no competing interests.

## Authors' contributions

SG was responsible for study design, analysis and interpretation of the patient data regarding the adrenal disorder and the resection. ARM performed the histological examination of the tumour, and was a major contributor in writing the manuscript. All authors read and approved the final manuscript.
